# Eradicating syphilis? Alternative projects, failures, and infectious disease control in the French colonial empire (1916–1940)

**DOI:** 10.1017/mdh.2025.10048

**Published:** 2026-01

**Authors:** Guillaume Linte

**Affiliations:** MESOPOLHIS, CNRS, Aix Marseille University, Sciences Po Aix, Aix-en-Provence, France

**Keywords:** Syphilis, Colonial health, Arthur Vernes, Institut Prophylactique, Eradication, Infectious disease control, French colonial empire

## Abstract

The inter-war period was a time of mobilisation against syphilis in France and its colonial empire. The spread of the disease was perceived as a threat to the economic development of its colonies, particularly because of the labour shortages it might cause. In 1916, a new player appeared on the scene of the French efforts to control the disease: the Institut Prophylactique, founded by Arthur Vernes. Its project was nothing less than to eradicate the disease, and its activities in the colonies expanded significantly during the 1920s and 1930s. However, the Institut Prophylactique has been largely forgotten in the history of medicine. Although the project was a failure, this article shows that it played an important role in controlling syphilis, both in France and in its colonies. This historical study thus emphasises the importance of considering alternative and failed projects as part of the complex picture of health history.

## Introduction

After the First World War, syphilis was the subject of particular scrutiny in France due to its perceived threat to the necessary ‘regeneration’ of the nation (i.e. to give a new demographic impetus to a country devastated by war). In addition to a declining birth rate attributed to miscarriages and infertility, the spectre of ‘racial degeneration’, embodied by hereditary syphilis (*hérédosyphilis*),^1^ hung over the country’s future. This period of mobilisation against the ‘venereal peril’ saw screening develop, health prevention intensify, and new actors emerge.^2^ In the French colonial empire, syphilis also became an increasingly important health issue due to its negative impact on demography. The colonial administration perceived the spread of the disease as a threat to the economic development of the colonies, potentially causing a labour shortage. In Africa, for example, the racist expression ‘*Faire du nègre*’ (i.e. promoting births to renew the African workforce) became a widespread slogan in health and political discourses as well as in the media.^3^ It was a way for the coloniser to express demographic anxiety and its solutions in a context shaped by racial discrimination.

It was in this context that a new player emerged at the forefront of the French effort to ‘fight’ syphilis: the *Institut Prophylactique* (in English, Prophylactic Institute; hereafter referred to as IP). Founded in Paris by Arthur Vernes, its project was nothing less than to eradicate the disease. As Vernes himself put it, the aim was to achieve the ‘extinction of syphilis’. During the 1920s and 1930s, the IP’s activities expanded significantly across France and its colonial empire. Despite this, the IP has been largely forgotten in the history of syphilis control.^4^ Although it had a significant influence during the inter-war period, the project was ultimately a failure. A failure not only in terms of eradicating the disease, but above all in terms of successfully imposing a new approach for treating and monitoring patients. Whether in France or the colonies, Arthur Vernes’ Institute was unable to convince with its methods and never managed to supplant its main rivals, the Institut Fournier and the Institut Pasteur.^5^ Less well known than Pasteur, the former was founded in Paris in 1923, named after France’s most famous syphiligrapher, Alfred Fournier (1832–1914). During the inter-war period, it expanded into the main cities of metropolitan France, with the mission of controlling sexually transmitted diseases.

The issue of syphilis in colonial empires has been the subject of significant re-evaluation in recent decades. In her seminal work *Curing Their Ills* (1991), Megan Vaughan dedicates a chapter to this question in East and Central Africa, in which she underlines the importance of the representation of African sexuality, regarded as uncontrolled, in the British colonial authorities’ approach to STIs. Furthermore, she demonstrates that a change occurred between the two world wars, when the primary concern shifted to birth rates and demographics. As she states, ‘The war had brought about a situation in which sexually transmitted disease was perceived as a problem for the Empire as a whole, white and black, with similar causes and similar solutions’.^6^ Her work also demonstrated how the concept of epidemic had been applied to syphilis in Uganda, thereby emphasising the socially constructed nature of its application to ‘venereal diseases’ in the early twentieth century.^7^ As with other issues in health history, the British Empire has been the focus of most historians’ attention regarding the history of syphilis. The issue of prostitution was examined in relation to race and gender. Philippa Levine, for instance, has demonstrated that STIs control policies from the Victorian era to the First World War were ‘ostensibly implemented to protect soldiers from the depredations of sexually transmissible diseases’.^8^ From a longer term perspective, Katherine Paugh examined the impact of knowledge dissemination on the representation of diseases in the Atlantic world and emphasised the importance of considering syphilis and its associations with other treponematoses (e.g. yaws and bejel) over a long period of time (from the eighteenth to the twentieth centuries).^9^

Other colonial empires have received less attention in relation to the history of syphilis, but this has changed in recent years, with historical works covering mainly the period from the First World War to the early 1960s. The Portuguese case, for example, has recently attracted significant renewed interest. Samuël Coghe has demonstrated how syphilis was incorporated into imperial ‘population policies’ in Angola with regard to infant mortality and maternity policy.^10^ In relation to Portuguese Guinea, Philip Havik emphasised a dual approach adopted by the colonial administration with regard to the issue of STIs: ‘a repressive one towards African women depicted as sex workers and disseminators of disease, and a paternalistic discourse reserved for women as (purportedly inept) progenitors of future generations’.^11^ In the context of the Belgian colonial empire, the cases of Rwanda and Congo have been examined from the general perspective of health policies, especially during the inter-war period,^12^ as well as in relation to the role of policies against ‘venereal diseases’ in colonial urban planning.^13^

The years following the HIV/AIDS outbreak stimulated historical research on STIs during the colonial era.^14^ In relation to the French colonial empire, a historiographical renewal occurred in the early 2000s, emphasising the continuities between the colonial period and the AIDS years,^15^ while providing a substantial number of case studies. This research provides a valuable insight into the multifaceted nature of syphilis’ history within the context of the French colonies. For instance, prostitution constituted a salient element of the issue in North Africa and Indochina, where specific health policies and regulations were introduced.^16^ The situation was quite different in sub-Saharan Africa. While Senegal was the main centre where efforts were made to control STIs, with major medical facilities (such as the Roume Polyclinic in Dakar),^17^ in other regions the rules put in place did not fit local realities but were based on a model similar to that of prostitution in France. In Cameroon, for example, there were few prostitutes who were officially registered, while occasional prostitution took place outside the framework defined by the colonial administration.^18^ A similar observation can be made in Upper Volta (Haute-Volta), emphasising the French authorities’ lack of understanding of the cultural practices of colonised African societies.^19^

Finally, the issue of syphilis was also addressed from the perspective of the very definition of the disease in a colonial context, particularly from the early twentieth century onwards. Recent studies have highlighted the role played by colonial medicine in distinguishing between different forms of the disease, defining ‘Arab syphilis’ in North Africa,^20^ ‘Tahitian syphilis’,^21^ and ‘exotic syphilis’ in Africa and Asia.^22^ In the latter case, this was a significant nosological shift, making syphilis a ‘benign’ dermatological disease for colonised populations (only). This redefinition allowed the colonial administration to minimise the health problem and justify its negligence, but it also led to the development of a particular therapeutic practice: *blanchiment* [lit. ‘clearing’],^23^ which consisted of a superficial treatment of syphilis designed to eliminate only the dermatological symptoms without attempting to cure the patients. It was seen as a way of limiting the spread of the disease by rendering individuals temporarily non-contagious.^24^

It was within this context that the IP emerged onto the colonial scene, introducing ideas that fundamentally opposed standard practices for treating syphilis in Africa and Asia, particularly *Blanchiment.* Its approach – without meaning that racism was absent among its doctors and health professionals – differed from the dogmas that dominated colonial medicine, in which colonised populations were considered to be insufficiently civilised to be treated in the same way as Europeans, at least without coercion. Arthur Vernes’ therapeutic methods encompassed a comprehensive approach to treatment for all patients, irrespective of their origin, with the overarching objective of eradicating syphilis on a global scale. The colonial world, as Helen Tilley has noted in her work on Africa as a ‘living laboratory’,^25^ provided a conducive environment for the emergence of such initiatives. Although the Institut Fournier may have overshadowed its counterpart in France, this did not result in any colonial ramifications. Consequently, the IP regarded the empire’s territories as a strategic opportunity for developing its own methods and establishing its leadership in the field of syphilis control.

This article analyses the development of the Institut Prophylactique through its colonial expansion during the inter-war period. It shows that, although it was unsuccessful in its goals, and its methods were not widely adopted, it nonetheless played an important role in controlling syphilis in France and the colonies. The history of mobilisation against the disease in metropolitan France after the First World War cannot be understood without considering its colonial empire, and vice versa. Drawing on this case study, I argue that alternative, abandoned, and unsuccessful projects to control infectious diseases played a crucial role in shaping colonial health policies and healthcare practices. Examining such cases is no less enlightening than studying the history of more successful or prevailing therapeutic and prophylactic approaches.^26^ By studying the history of the IP, I demonstrate that the choices made to control syphilis in the French colonial empire were challenged in the inter-war period and that alternative proposals were on the table. The IP not only proposed a new therapeutic strategy but also a different approach to caring for colonised patients – compared with the prevailing approach of superficially treating syphilis through *Blanchiment.* ^27^ I therefore emphasise the importance of considering alternative and failed projects as part of the complex picture of health history and infectious disease control.

In this article, ‘alternative’ refers to the competition between health-related medical and political projects not only in the colonial empire but also in metropolitan France. It does not pretend to cover the ‘alternative’ dimension arising from the projects or resistance strategies of colonised populations, or the issue of hybridisation or medical pluralism.^28^ This study aimed to provide an insight into the plural dimension of colonial medicine and health policies during the inter-war period, as well as situating the history of the IP within the broader context of infectious disease control strategies during the colonial era.

This research is based on analysing and cross-referencing a large corpus of archives and printed sources. First, it is based on a comprehensive study of the *Archives de l’Institut Prophylactique*, the institute’s journal, which, to date, has received little attention from historians. Second, relevant material has been gathered from a vast corpus of archives, including the French National Archives (AN), the French Overseas National Archives (ANOM), the National Archives of Senegal (ANS), and the archives of the Academy of Medicine in Paris (AMP). Third, it draws on studies published over the last twenty years on the history of syphilis in the French colonial empire, giving a specific attention to recent historiography about colonial therapeutic strategies, particularly mass medicine and *blanchiment.* ^29^

After tracing the rise of the IP and the principles of its objective to achieve the ‘extinction of syphilis’ (part 1), I highlight the history of its colonial expansion, both from the point of view of its overseas development (part 2) and the strategy of adapting its practices to the colonial context (part 3). I then emphasise the importance of an approach on a local scale to understand how it developed and its challenges in the colonial context, taking Indochina as a case study (part 4). Finally, I examine the reasons for the IP’s demise in the 1930s, stressing the importance of the colonial outlet for its success (part 5) as well as the failure of its colonial expansion (part 6).

## The Institut Prophylactique and the Vernes method for the ‘extinction’ of syphilis

Founded in 1916, the Institut Prophylactique was a leading institution for controlling syphilis during the inter-war period in France. It was intended not just to offer treatment for syphilis, as many dispensaries and hospitals did this (such as the *Hôpital Saint-Louis* in Paris), but also to be the cornerstone of a project designed to lead to the ‘extinction of syphilis’.^30^ Trained in both medicine and microbiology, as an intern of the Paris hospitals between 1907 and 1912, Arthur Vernes specialised in dermatology and syphilology – a medical specialty dedicated to the study of syphilis.^31^ His thesis, ‘Introduction to the Study of Experimental Conditions for the Treatment of Syphilis’, which was released in 1913, was the first step towards the development of a new, ‘rational’ method of treating syphilis.^32^ This approach was based on the idea that the existing treatments offered to syphilitics were ‘blind’ because they were not supported by sufficiently accurate testing methods. According to him, the main weakness of the current techniques, including the Wassermann reaction,^33^ was that they did not enable monitoring of how the treatment influenced the evolution of the disease.^34^ To remedy this situation, Vernes developed a new detection method, ‘syphilimetry’, generally referred to as the Vernes test or reaction. Using a new measuring device, the photometer [*photomètre*], this method not only indicated whether a person was infected but also measured the degree of infection using a graduated scale. The benefit of measuring the level of infection was that responses to different treatments could be recorded. This facility enabled treatments to be identified as unsuitable for a specific patient and thus modified to achieve the cure.

Furthermore, Vernes advocated for what he called the ‘eight-month rule’, according to which an individual would be declared to have recovered from syphilis only after eight months of negative results using the photometer.^35^ He maintained that this protocol constituted the first rational method of treatment of syphilis and the only one that could assert with confidence the definitive cure of a patient, since the Wassermann reaction failed to predict relapses. In the words of Vernes in 1929, ‘What remains extremely dangerous, armed as we are today [against syphilis], is much less the microbe itself than the doctrines that allow it to stay in place.’^36^ Unsupervised treatments – meaning all those uninformed by syphilimetry – were considered to be responsible for the ‘extension and immensity of the devastation’ of syphilis.^37^

In short, what was called the ‘Vernes method’ during the inter-war period consisted of three elements: a new and quantitative approach to the extent of syphilitic infection, syphilimetry; a new device to achieve this, the photometer (or ‘Vernes test’); and a new approach to treatment involving long term follow-up after the test became negative, with the eight-month rule. The early days of the IP and syphilimetry were also supported by a seminal corpus written by Vernes for physicians. This corpus comprises an *Atlas de syphilimétrie* [Atlas of Syphilimetry], published in 1920, and five fascicles edited between 1922 and 1926.^38^ This set of texts, containing the research carried out after his thesis, formed the basis of the ‘Vernes method’. It synthesised both the technical aspects (i.e. the use of the photometer and the realisation of curves indicating the infection level) and the therapeutic doctrine (the need for a treatment combined with a close serological follow-up and evidence of a cure over eight months).

Located in Paris, the Institute grew rapidly during the 1920s, supported by public subsidies from the State and local governments (*départements* and municipalities). The total number of consultations rose from 10,753 in 1916 to 239,284 in 1929. By that time, the IP claimed to have installed its screening equipment in over a hundred locations in France and forty-one foreign countries.^39^ This success is also due to the ability of its founder. Vernes succeeded in gathering support for his project from both the scientific community and the political world. Emile Roux, the director of the Institut Pasteur, agreed to be associated as Honorary President. At the same time, the IP was placed under the high patronage of the President of the French Republic, Gaston Doumergue, and the former President of the Republic, Alexandre Millerand.^40^ Vernes also received the support of influential people in the field of culture, including Eugene Brieux, a member of the *Académie Française* and author of *Damaged Goods* [‘*Les Avariés*’], a theatrical play about the effects of syphilis, which had reached an international audience in the early twentieth century.^41^ The IP continued to grow in the early 1930s, with 340,000 consultations in 1932 and 2,900,000 francs in subsidies from the State and local government.^42^

The IP’s growing prominence during the inter-war period in France was also reflected in the creation, in 1929, of a journal devoted to its life and field of interest: *Archives de l’Institut Prophylactique* [Archives of the Institut Prophylactique]. Releasing four issues each year until 1940, when it ceased publication, the journal was an essential tool in promoting syphilimetry and the Institute’s activities, as well as those of Vernes himself, who was the author or co-author of approximately half of the papers it published. Moreover, most articles were written by members of the IP or doctors convinced by Vernes’ ‘discoveries’ and methods. They were usually very flattering towards the founder of syphilimetry. Beyond disseminating the new approach to testing and treating syphilis, the journal’s founding was clearly aimed at making the IP a leading health establishment in France, just as the Institut Pasteur then was. However, to achieve this latter aim, Vernes’ best argument was to promise a spectacular and enduring result: the eradication (‘extinction’) of syphilis.

## The colonial expansion of the Institut Prophylactique

In the 1920s, the IP developed a strong interest in applying syphilimetry in the colonies. This contrasted with the Institut Alfred Fournier (founded in Paris in 1923), which paid little attention to France’s overseas empire. The idea did not seem to originate from Vernes but rather from his close collaborator, Marcel Léger. Born in Guadeloupe and trained at the Naval Health School of Bordeaux, Léger had significant experience of medicine in the colonial context. He had participated in a campaign against yellow fever in Sudan in 1902, and he practiced in Tonkin from 1909 to 1911 before completing his training in 1913 at the Ecole du Pharo, a new medical school founded in Marseille for the training of colonial health officers. He then held several prominent positions between 1916 and his retirement in 1925, as the director of the Health Service in Guyana, an assistant professor at the Ecole du Pharo, and then director of the Institute of Biology in Dakar, which became a branch of the Pasteur Institute under his leadership.^43^ Léger was quickly convinced by the Vernes method and widely promoted the benefits of extending syphilimetry to the colonies.^44^ After his retirement, he became one of the most active members of the institute, both through the *Archives de l’Institut Prophylactiques* and intensive activity within the *Société de Pathologie Exotique* [Society of Exotic Pathology], of which he became vice president in 1929.

From the middle of the 1920s, the IP began to expand its activities in the French colonies. The first colonial service was founded in Saigon (Indochina) in 1926, with a second opening in Tananarive (Madagascar) a year later.^45^ The establishment of the ‘Vernes laboratories’ in the colonies accelerated during the following years in the form of independent establishments or as complements to existing medical structures. By 1930, six Vernes laboratories had been established in the colonial empire. In addition to the independent institutes in Saigon and Tananarive, syphilimetry facilities were created at the Bacteriological Institute in Pondicherry, the Institute of Hygiene in Cayenne, and the *Hôpital Principal* in Dakar, in addition to a dispensary in Algiers.^46^ The implementation of syphilimetry subsequently accelerated, especially in Madagascar, led by the Medical Officer René Trautmann, who was also the director of the colony’s Institut Prophylactique. In 1931, the island had no fewer than five laboratories, while six others were already in the pipeline.^47^ The diffusion of the Vernes method soon concerned all parts of the colonial empire: By 1935, twenty centres of syphilimetry had been established in ten different colonies or territories under the mandate of the League of Nations.^48^ However, it should be noted that the nature of a ‘Vernes laboratory’ is particularly difficult to grasp. It may have been a genuine medical institution that attempted to follow the Vernes method, as was the case in Tananarive and Madagascar, or it may have been a small facility or an annex to a bacteriology laboratory. Due to the limited historical data available, the exact nature of some of these laboratories remains unclear.

As for the IP’s development in France, Vernes and Léger succeeded in constructing a network of political and scientific support. They recruited the Ministry of Colonies to their cause, counting on the support of the *Sous-Secrétaire d’Etat* [State Under-Secretary] Alcide Delmont, who attended the Institute’s annual General Assembly on 20 December 1929.^49^ Shortly afterwards, on 7 February 1930, Vernes was invited to give a talk to the *Académie des Sciences Coloniales* [Academy of Colonial Science], of which Léger had been a member since 1923. In his speech, Vernes advocated for the organisation of the extinction of syphilis in the colonies:

The question of whether the extinction of syphilis could be organised in our colonies depends on the point of view from which we place ourselves and on the guarantees that could be obtained concerning the methods we use to achieve it.

It would be vital that the thing could be done. Far from being, as one might think, inexhaustible reservoirs of men, our colonies are becoming more and more decimated. Syphilis is one of the fundamental causes.^50^

In his presentation, Vernes stressed the broad necessity for training colonial health officers – and the ‘native’ [‘*indigène*’] staff – in the methods of syphilimetry. He also used an economic argument, deeming that the blind distribution of treatments weighed heavily on the colonial budget: ‘The costs of the first installation and the operating costs are rapidly recouped by the economy of the results. […] Indeed, by preventing the use of blind treatments and the waste of medicines, syphilimetry is, from the budgetary point of view, exceptionally economical.’^51^ Following this communication, the *Académie des Sciences Coloniales* decided to support the IP through a ‘wish’ addressed to the Minister of the Colonies: ‘In addition to the dispensaries, the number of which should be multiplied, it is necessary to create serology laboratories in all the centres, equipped with the Vernes syphilimetric equipment, which has already proved its worth in Dakar, Saigon, Tananarive, and Pondicherry.’^52^

On 13 August 1929, the Superior Council of Health of the Colonies created a Consultative Commission for the Prophylaxis of Venereal Diseases in the Colonies [*Commission Consultative de Prophylaxie des Maladies Vénériennes aux Colonies*]. Its mission was ‘to study the appropriate measures to intensify the prophylaxis and treatment of venereal diseases in the colonies’ and to ‘coordinate these measures with those taken in the metropole under the direction of the National Office of Hygiene’.^53^ The IP was actively involved in this Commission, which was composed of twelve members, some of whom were influential doctors in the field of colonial health (e.g. Séverin Abbatucci) or in the control of venereal diseases (e.g. André Cavaillon, Head of the Department of Prophylaxis of Venereal Diseases at the National Office of Hygiene). It also included Vernes and Léger, as well as two prominent political supporters of the IP, the senator of *Côtes-du-Nord*, Pierre Even (the President), and the deputy of Guadeloupe – also a member of the Institute’s Administrative Council – Gratien Candace (the Vice President).

On 11 July 1930, Léger presented a report to the Commission entitled ‘The fight against syphilis in the native population of the colonies’.^54^ In it, he stressed the crucial political and economic stakes of syphilis control:

The fate of France is tied to that of its colonies. To supply our military contingents with men, to develop our overseas wealth, and to take advantage of it, it is essential to protect human capital by eliminating or attenuating the endemic disease that most affects its vitality. The development of our vast colonial empire is only possible on condition that we attack with energy the scourge that immolates millions of individuals every year, that bastardises individuals physically and mentally, that kills the child at birth or even in the mother’s womb.^55^

According to Léger, implementing the Vernes method is necessary to allow ‘the organisation of a rational fight against syphilis’. Outlining an upbeat assessment of the first applications of syphilimetry in Madagascar, Senegal, Indochina, Pondicherry, and French Guiana, he urged the Commission to ‘pursue and coordinate efforts to ensure the extinction of syphilis by the complete elimination of the infectious germ in syphilitic patients’.^56^ A year and a half later, in December 1931, a sanitary inspection mission in Guadeloupe was led by Léger and Even, supported by several ministries (those of the Colonies, Merchant Navy, and Public Health) and financed by the General Council of Guadeloupe.^57^ The report of the ‘Even-Léger mission’ was presented to the Commission on 29 January and 17 March 1931, which then approved the application of syphilimetry in Guadeloupe.^58^

## Colonising syphilimetry: Adapting materials and training ‘natives’

Despite the commitment of Léger and Vernes, the success of the colonial deployment of syphilimetry could not depend solely on lobbying in France. The presence of doctors who were convinced by the Vernes method and able to ensure its implementation in the colonial field was crucial. Two colonies quickly met these conditions: Tonkin, around the Institut Prophylactique of Saigon, directed by Dr. Nguyen Van Tung, and Madagascar, with the Institut Prophylactique of Tananarive led by René Trautmann. These two physicians played a decisive role in the deployment of syphilimetry in the French overseas territories. While the two centres they directed soon became the IP’s flagships in the colonies, they also played a critical role in training Indochinese and Malagasy medical staff to use the photometer. The existence of recently created medical schools made these colonies ideal places to reach a large public that could become enthusiastic practitioners of syphilimetry. In Madagascar, Trautmann’s influence achieved widespread dissemination of the Vernes method, promoted by official propaganda, such as in institutional films. The film *Symphonie malgache* [Malagasy Symphony] (1934) – which aims to highlight the development of health, economy, and tourism on the island – shows ‘one of the ten Vernes laboratories of the colonies’ and the training of medical students where syphilimetry is presented as an exemplar of the modernity introduced by colonisation.^59^

Training healthcare staff, particularly French and ‘native’ physicians and nurses, was central to the IP’s colonial strategy. The training was carried out in its building in Paris and different structures in France related to the IP’s overseas activities. In 1930, Vernes stated: ‘At the request of the Health Service of the Ministry of Colonies, we have already trained an interesting number of physicians of the colonial troops.’^60^ This investment in training was not specific to IP’s colonial interests; it was part of its national and international strategy, which consisted of training and disseminating information about syphilimetry to the maximum possible extent, including to all ‘physicians, pharmacists, serologists, students, and social nurses’. For this purpose, its laboratories opened their doors to anyone who took an interest in them:

The laboratories are open, free of charge, every day from 9 a.m. to 12 p.m. and from 2 to 5.30 p.m.. The practical demonstrations are led by a physician and a serologist. The trainees practice on small operations, as often as they want, and are comprehensively introduced to the use the photometer.

In addition, from 5.30 p.m. onwards, they attend the central dispensary consultation, which takes place every day. There, the doctors provide their patients with useful explanations.^61^

**Figure 1.**
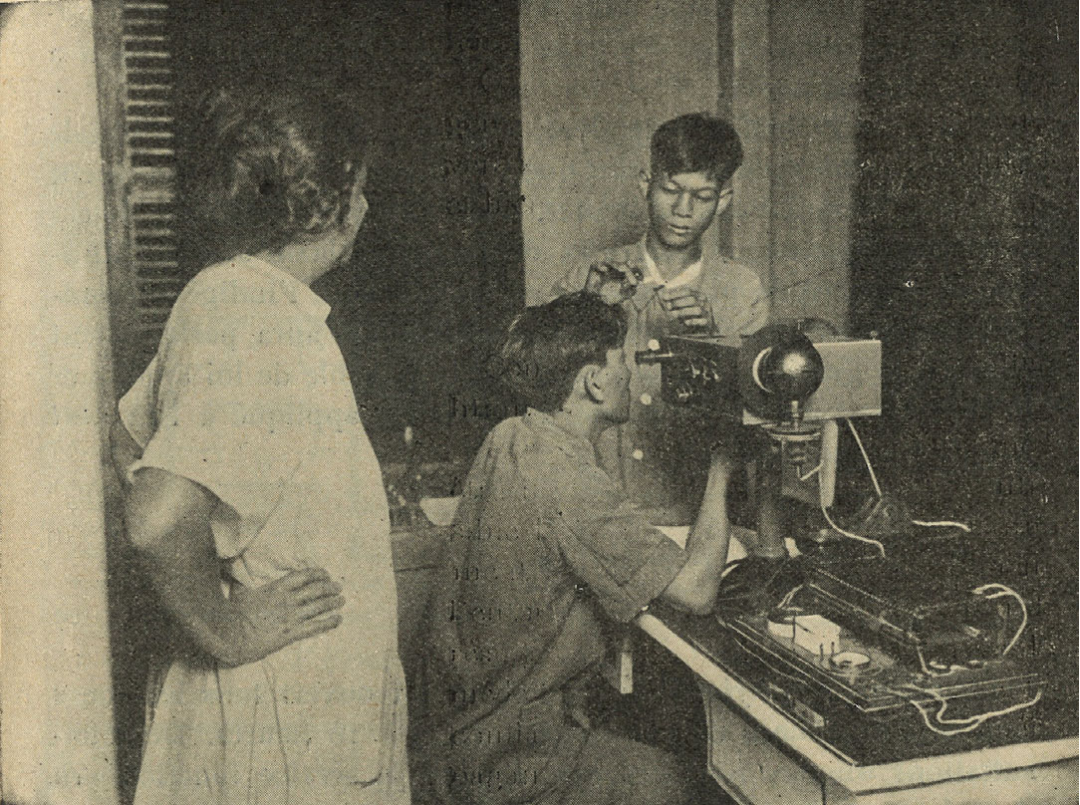
‘Native nurses being trained to use the photometer’, Archives de l’Institut Prophylactique, II, 1 (1930), 19. (photograph: the author.)

The Institut claimed 277 trainees during 1930,^62^ ‘who spent between 10 and 90 days in the services of the Institut Prophylactique’, as well as 300 occasional visitors. Demonstrations and lectures were also regularly organised for health students: ‘In short talks, the essential principles of syphilimetry were explained to them each time.’ For example, on 18 May 1930, a group of ten students from the School of Colonial Practice [*Ecole de Pratique Coloniale*] were welcomed. Conferences were also organised outside the Institute, such as one in Marseilles, on 11 June 1930, for the students of the School of the Colonial Health Service [*Ecole d’application du Service de Santé du Corps Colonial*].^63^ The training of health professionals from colonised populations was no less important than that of colonial officers. This training took place in France for a minority – such as Nguyen Van Tung (later the head of the Institute of Saigon) – but mainly in the major syphilimetric centres of the colonies. In these centres, nurses and health auxiliaries formed the bulk of the trained contingent. This is particularly emphasised in the articles of the *Archives de l’Institut Prophylactique.* The photograph below (Figure 1) illustrates how the IP promoted its training activities in its journal. It shows a French female nurse training two Indochinese male nurses in the use of the photometer, thus conveying an idea of medical progress through the teaching of the latest laboratory techniques. Besides the development of the Vernes method overseas, the journal aimed to highlight the Institute’s contribution to the educational and ‘civilising’ work of France in the colonies.

The extension of syphilimetry in the overseas empire could not be achieved by simply translating equipment and practices from the metropolitan model. The photometer and handling of serum had been designed for use in France, with its easy access to electricity and temperate climate. These conditions were not satisfied in some colonies, especially outside the main urban centres. At the end of the 1920s, Vernes and Robert Bricq, co-designer of the photometer, in collaboration with Trautmann, worked on the adaptation of the required material for syphilimetry and explained their solutions in an article of the *Archives de l’Institut Prophylactique*:

In order to satisfy all the present and future colonial needs, we had to make some modifications to the equipment. They make it possible to operate in localities without an ice factory or an electric factory. It should be noted that, in the colonies, gas plants are non-existent and, in those cities having an electric plant, the electricity is often irregularly distributed, sometimes cut off during a part of the day and on public holidays.

We present here the equipment, adapted for colonial practice, that various manufacturers have realised, either on their own or based on our instructions.^64^

**Figure 2.**
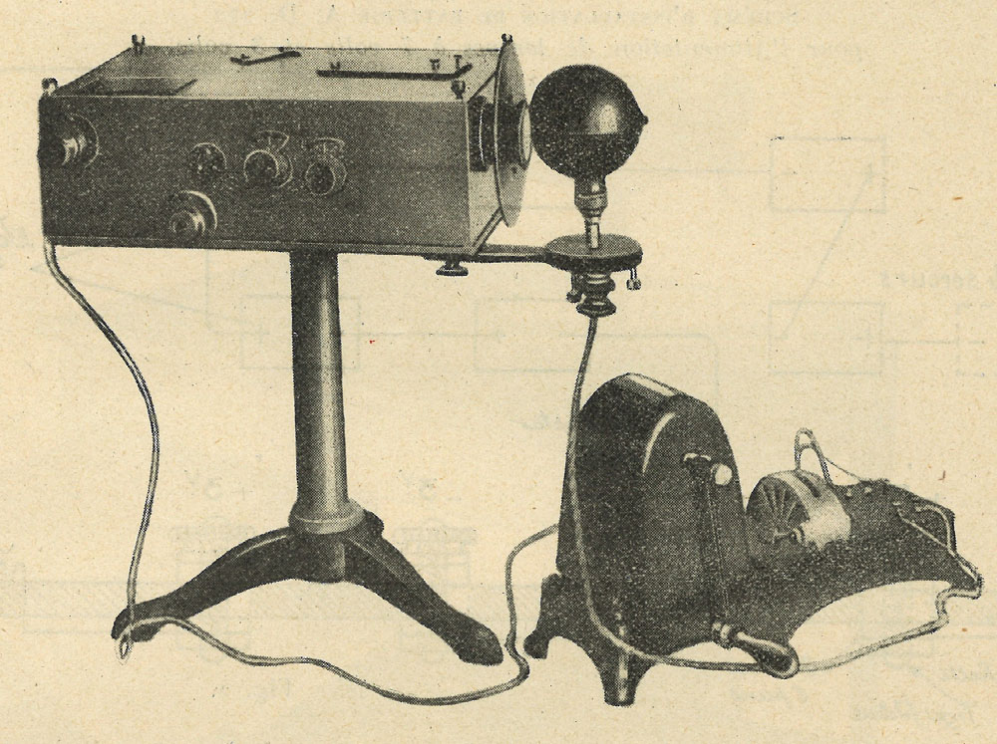
Photometer combined with a Pathé-Baby. Archives de l’Institut Prophylactique, II, 1 (1930), 72. (photograph: the author.)

The technical difficulties were numerous, including lighting of the photometer, functioning of the mixers and centrifuges, keeping the samples cool, and maintaining a constant temperature in the water bath. For the lighting of the photometer, for example, batteries were chosen as the solution. The battery was in a box fitted out by the ‘Maison Jobin et Yvon’, with a capacity of 200 hours, or (according to the authors) one year of functioning in a colonial dispensary. Nevertheless, it remained particularly cumbersome and challenging to transport. Vernes, Bricq, and Trautmann proposed the adaptation of a portable film projection device for less accessible posts or mobile teams; this device was commercialised in 1922 by Charles Pathé and named the Pathé-Baby. Several models were developed, each allowing ‘the projection of animated images in homes, within small communities in cities, or in the countryside’.^65^ Its advantage for syphilimetry was its ability to produce light using a crank, without requiring electricity; the crank enabled the photometer’s interior to be illuminated to read the results. According to Léger, this equipment was trialled in Madagascar as early as 1930 (see Figure 2).^66^

Colonising syphilimetry through adapting the necessary equipment to local conditions was particularly important in the IP’s work. The institute wished to place these innovations in the framework of modernising the colonies, showing how the fight against syphilis was integral to developing infrastructures and economy. At the International Colonial Exhibition of 1931, held in Paris at the Grand-Palais, the IP hosted a stand to praise its achievements. This highlighted the devices created or adapted for the colonies but also presented a complete set of propaganda material to introduce the public to its activity overseas. It included ‘photographs of various colonial Instituts Prophylactiques, statistics, charts of patients, etc.’, the list of the ‘36 overseas services existing at the time of the Colonial Exhibition’, and ‘a planisphere showing the extension of syphilimetry in various parts of the world, and in particular in the colonies’.^67^

## Global project vs field implementation: The case of Indochina

The local scale (i.e. a colony or a regional group of colonies, such as Indochina here) helps to understand the actors involved and the issues at stake in the IP’s colonial expansion. In this section, I focus on a case study that is particularly revealing of the situated dynamics that explain the limits of its development and its failure to establish itself as the main player in the control of venereal diseases in the colonies: Indochina.

Indochina was the first colony to welcome the Vernes project. The service opened its doors in Saigon (Cochinchina) on 1 May 1926, in a building adjacent to the Municipal Polyclinic.^68^ The creation of the Saigon Institute was partly intended to meet the obligations associated with the signing of the Brussels Agreement of 1924. In a ‘Report on social hygiene in Indochina’, produced by the General Inspection of Health and Medical Services and dated 1926, its role is clearly explained:

A Prophylactic Institute for venereal diseases is organised and operates in Saigon; in 1926, it received and treated 3,777 patients. In compliance with the Brussels International Arrangement […], in an application before the letter of its terms to the colonies, it is open and free of charge to seafarers; the maritime authorities have been notified of this innovation.^69^

The document also states that similar facilities were ‘under consideration’ in other parts of the colony. The Saigon Institute was a great success from the outset. Open from 7 to 10 a.m., Monday to Saturday, consultations were provided by Indochinese physicians trained at the Hanoi Medical School.^70^ Its director, Nguyen Van Tung, was also trained at the Hanoi Medical School, as well as in metropolitan France at Paris’ Faculty of Medicine, Institute of Colonial Medicine, and the IP headquarters.^71^ He practiced at Hospital Tenon in Paris, preparing a thesis under the supervision of Edouard Jeanselme,^72^ one of the most influential dermato-venereologists in France. Nguyen Van Tung reflected the IP’s strategy of relying on physicians from the imperial regions where it intended to expand. The IP of Saigon, founded in 1926, received many patients during its first years of activity. The colonial press relayed the enthusiasm of the colony’s governor at the opening of an institute ‘offering patients the most recent methods of diagnosis and treatment’.^73^

The Saigon Institute took on a new dimension when, on 8 April 1930, it inaugurated its new building. In a series of speeches delivered before Governor Cognacq, Nguyen Van Tung announced his intention to ‘organise a veritable crusade’ against syphilis: ‘Unmasking the disease, educating the patient, convincing him of the need to follow a long and regular course of treatment, not aggravating his case, curing him quickly; these are the fundamental rules governing the operation of the Institut Prophylactique.’^74^

The Saigon Institute continued to grow its activity over the years, supported by the colonial administration, which increasingly relied on its efforts. In 1933, the Inspector General of Hygiene and Health requested the opening of an evening consultation to complement the one that occurred in the mornings.^75^ At the time, venereal diseases – whose prevalence was constantly rising – were seen as a significant threat to Indochina. Consequently, on 2 December 1933, the Governor General set up a ‘Commission for the prophylaxis of venereal diseases in Indochina’,^76^ which held its first meeting in Hanoi on 8 January 1934. The commission comprised a dozen physicians and colonial administrators, including a fervent defender of syphilimetry, François Sorel. The military authorities were one of the main driving forces behind the commission’s creation, noting ‘the spread of venereal diseases among the troops’. They were worried that, in the event of a military operation, ‘40 or even 60%’ of their troops would be unavailable due to venereal disease.^77^ Nevertheless, the commission’s aim was broader, deciding on action on colonial society, as a whole, to reduce the sources of contagion. Article 3 of the decree establishing the commission specifies its objective: ‘This Commission will look for appropriate prophylactic measures to control the development of venereal diseases in the Colony’.^78^

The role of the IP in the fight against venereal disease was a key issue in the commission’s discussions. On a colony-wide scale, the dissemination of the Vernes method was fervently defended by Sorel. However, the injunction to give syphilimetry a significant role in anti-venereal policy originated directly from metropolitan France and the Ministry of the Colonies. As early as 16 March 1932, the Minister had asked the government of Indochina to ‘multiply the number of prophylactic institutes’.^79^ Sorel recalled this during his speech at the commission’s first meeting and advocated the installation of syphilimetry equipment in all centres devoted to treating venereal diseases. Apart from those at the Institute of Saigon, there was only one photometer in Hanoi at the beginning of 1933, thanks to ‘a private initiative’.^80^ However, discussions within the commission mainly focused on policing prostitution and educating soldiers. On 2 May 1934, the commission’s president, Auguste Tholance – also the *Résident Supérieur* in Tonkin – reaffirmed that ‘In principle, the construction of a Prophylactic Institute in Hanoi has been planned.’^81^ This second session was also followed by the expression of ‘wishes’, including the creation of ‘treatment centres similar to the Institut Prophylactique’.^82^

Although the context seemed favourable for the development of the Vernes institutes in Indochina, their field implementation was hindered, especially due to the opposition of physicians and administrators occupying key positions in the colony. In the summer of 1934, the Director of Health in Tonkin, De Raymond, considered that the current facilities already fulfilled the functions of an Institut Prophylactique. He saw syphilimetry only as one testing method among others, one ‘which is not indispensable […] as there are other equally effective means of control’.^83^ He criticised the desire to multiply the number of institutes, saying that, outside of large towns, they were ‘not necessary and not adapted to native settlements, nor even to garrisons’. He emphasised the cost to the colony of such a deployment, ‘which would involve expenditure on buildings, personnel, and equipment out of all proportion to the importance of the centres that would be equipped with them’.^84^ De Raymond defended his views at the commission’s third meeting on 27 September 1934, adding that Indochinese patients were not sufficiently ‘persevering’ to follow a long-term treatment program.^85^ A racist commonplace widely shared by French healthcare professionals between the wars. This meeting was punctuated by a vigorous exchange between De Raymond and Sorel, highlighting the difficulties faced by the IP in explaining the specificities of their approach compared to conventional treatments. Tholance interrupted the discussion to ask what the Vernes method was, even though the creation of an IP in Hanoi had been planned for more than two years. Sorel complied, pointing out that ‘the great advantage of this method’ lies in the fact that treatment can be controlled right up to the point of recovery, ‘whereas in the old methods, patients continue to be treated, once cured, by overloading them with mercury or potassium iodide’. Sorel clearly struggled to convince, while De Raymond reiterated his opposition, pointing out that the use of the Vernes test ‘has not even been generalised in France’s venereology services’.^86^

No new institutes were created in Indochina, except for a Vernes laboratory in a military garrison in Tonkin. Other options were favoured, such as a project to establish a reserved district for prostitution in Hanoi. At the end of 1936, the Minister of Colonies wrote to the Governor General of Indochina, René Robin, to express his dissatisfaction. The Minister stated that ‘the impetus given in Indochina to the fight against the venereal scourge has been clearly insufficient’, pointing out that the order concerning the creation of an Institut Prophylactique in Hanoi had not been respected: ‘Even in the large centre of Hanoi, where a very precise plan had been drawn up, the measures taken to date have been totally insufficient’.^87^ Following the Minister’s reprimand, Robin lashed out at Dr. Hermant, Inspector General of Hygiene and Public Health, asking him to assess the situation and answer the question: ‘Who is responsible?’ In his report, Hermant reveals how little progress and investment had been made in the anti-venereal control effort in previous years, except in Cochinchina:

[Cochinchina] had made sacrifices in advance from its own budget for a prophylactic institute, which continues to be improved by its own resources – 60,000 francs since 1932 – and in 1936, the town of Cholon used its own resources to build a polyclinic in which a venereal disease consultation adds a decentralised centre to the Saigon Institut Prophylactique for the ambulatory treatment of venereal patients.^88^

Holding a unique position in the Indochinese health system thanks to its close collaboration with the IP, Cochinchina was clearly the flagship of the anti-venereal effort in Indochina. The budget allocated there for 1935 was 1,675,668 francs (including 418,000 for the Institut of Saigon), representing around 62% of the total expenditure for Indochina as a whole. In comparison, Tonkin spent only 494,480 francs on venereal disease control in the same year.^89^ This imbalance was all the more significant given that Tonkin had almost twice as many inhabitants (8,700,000) as Cochinchina (4,616,000), of a total population estimated at 23,030,000 in Indochina.^90^ Furthermore, the establishment of the IP had a measurable influence on how patients with venereal diseases were treated. The number of consultations was much higher than in other provinces, allowing patients to be monitored over a more extended period. On average, a syphilitic patient was seen nine times a year in Cochinchina, compared with just five consultations in the other provinces (see Table 1). Hospitalisation was also much more frequent.^91^ These figures, while testifying to better care for syphilitic patients in Cochinchina, might also have worked against the Vernes method by highlighting the financial cost of its program.

**Table 1.** State of the VD control in 1935, according to Dr Hermant (ANOM, RST NF 3856)

Province	Population	Budget (francs)	Patients treated for VD	Total number of consultations	Days of hospitalisation for VD	Average consultations per patient
Annam	5,656,000	266,716	73,428	179,570	46,679	2.4
Cambodia	3,046,000	205,030	9,531	47,655	40,567	5.0
Cochinchina	4,616,000	1,675,588	63,973	560,217	189,480	8.8
Laos	1,012,000	35,900	3,876	19,380	5,985	5.0
Tonkin	8,700,000	497,480	21,054	101,752	53,912	4.8

Figures aside, it seems the IP benefited from a flattering reputation in Saigon. Its director, Nguyen Van Tung, embodied a new kind of modernity, both in terms of his background as an Indochinese physician and his modern approach to medical practice and teaching. In April 1939, for instance, the colonial newspaper *L’Echo Annamite* lauded his initiative in commissioning anti-venereal propaganda films from France: ‘The sympathetic Dr Nguyen-van-Tung [sic] has commissioned films in France designed to popularise the essential notions required to warn young people against the ravages of venereal diseases. This is an excellent initiative.’^92^ In 1941, writing in the same newspaper, Nguyen Van Tung praised French medical action in Indochina. However, he highlighted in particular that of the Institute of Saigon, which, in the midst of the Second World War, ‘applied essentially French diagnostic and treatment methods, such as those used at the IP of Paris’.^93^

In Indochina, the IP offered a new, ambitious alternative for controlling syphilis. Under the impetus of its director, it provided more comprehensive and attentive care for patients. However, despite his expertise, Nguyen Van Tung remained outside the debates on anti-venereal prophylaxis in Indochina during the 1930s. Due to opposition from physicians and administrators, even the support of the Ministry of Colonies was insufficient to foster the extension of syphilimetry in Indochina. This failure, despite its encouraging results, is symptomatic of the IP’s inability to establish itself as a sustainable alternative for the control of syphilis in the French colonial empire.

## The collapse of the Institut Prophylactique

The late 1920s and early 1930s saw the apogee of the Institut Prophylactique in France, as well as the rise of its colonial ambitions. However, the Institute spiralled into a severe decline after 1932. In a confidential note written in the summer of 1941, the Institut’s Council of Administration recalled this period of collapse:

But, from that date [1932], our Institute had to stop its progression and start to close services. Instead of being able – like any scientific institution – to hire workers who were solely dedicated to their task, it had to recruit them without possessing the means to pay them sufficiently, which meant that they had to find alternative sources of income; and the Institut Prophylactique had to struggle painfully, not only against syphilis, but against everything that prevents the fight to prevent it (indifference, ignorance, social prejudices, and medical prejudice against its curability); as if syphilis was a negligible enemy, as if our country had to be the last to benefit from the scientific discoveries that had led to the foundation of this Institute.^94^

The leading cause of the decline, according to the Board of Directors, was the disinterest and neglect of the fight against syphilis by the French government. This would have been particularly perceived through a stagnation of the allocated budget, which was no longer a priority. In a letter of 24 October 1941, addressed to Henri Gangardel, vice president of the IP, Vernes returns to these events and the progressive disinvestment of the State in a period of economic crisis: ‘The number of consultants started to decrease because we had to gradually close dispensaries, because the subsidies did not only fail to increase in proportion to the devaluation of the Franc, but decreased in real terms.’^95^ In addition, Vernes reported a chronic delay in payment of the subsidies. As a result, ‘end-of-the-month concerns forced us to question all necessary spending plans’. He provided detailed figures showing the spectacular fall in consultations from 1933 onwards,^96^ coupled with low and irregular subsidies. These insufficient finances caused the closure of services and the laying-off of staff.

Furthermore, despite some success, the Vernes test and syphilimetry failed to establish themselves as better alternatives to other diagnostic methods. During the inter-war period, the Bordet-Wassermann test was the preferred and most widely used test in Europe.^97^ Most of the time, the Vernes test was only employed to complement the Bordet-Wassermann test^98^ and was not intended to be used as part of the therapeutic programme prescribed by the IP. The fate of the Vernes test was partly sealed when, in 1931, Édouard Jeanselme published his *Traité de la syphilis* [Treatise on Syphilis], a seminal work in France in the 1930s. The first volume was dedicated to the history of syphilis and the evolution of diagnostic techniques. In this text, Jeanselme emphasised that reactions based on flocculation – such as the Vernes test – were no more accurate and ‘cannot currently replace the Bordet-Wassermann reaction’. In his opinion, their use was useful as a complement to provide additional accuracy. He therefore recommended using ‘the Bordet-Wassermann and flocculation reactions concurrently’.^99^ Attempts by Arthur Vernes and the IP to establish syphilimetry as the standard diagnostic method in France failed, and the Vernes test remained one among many others.

From a financial perspective, the situation continued to deteriorate during the Second World War and the Occupation. Although Maréchal Pétain was the new Honorary President of the IP, it received little consideration from the Vichy regime. By mid-October 1941, only 500,000 francs had been paid to the Institute, down from the state subsidy of 2,340,000 francs the year before.^100^ At this moment, its very existence was questioned. In early October, Vernes went to Vichy to plead his case to Pétain, who decided to support him.^101^ However, the resources allocated remained limited.

As a symbol of this collapse, the *Archives de l’Institut Prophylactique* also declined after Léger’s death in 1934. During the following years, the content of the official organ of the Institute became poorer, with a reduction of about one-third of the volume and a reduced scope. The war finally seems to end the slow agony of the journal’s demise since it ceased publication in 1940, with two volumes entirely written by Vernes and reporting on the action of the IP since its creation – the second volume covering the last nine months.

## Saving the IP through the colonies? Arthur Vernes in French West Africa (1938–1939)

**Figure 3.**
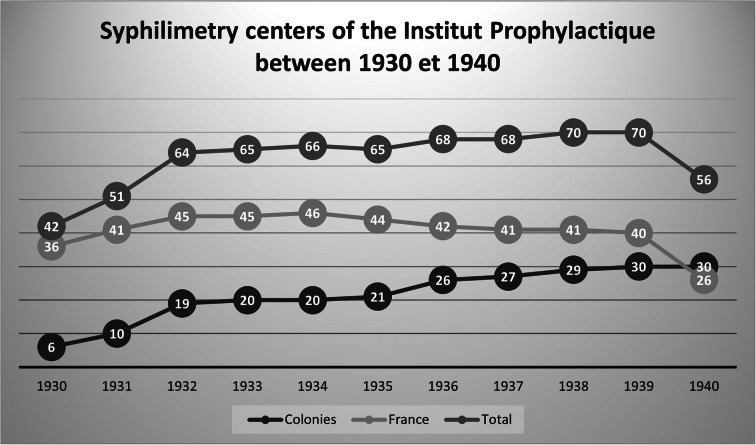
Syphilimetry centres of the Institut Prophylactique between 1930 and 1940. (graph: the author.)

In the colonies, unlike metropolitan France, the institute continued to expand in the 1930s (see Figure 3) even if it experienced a slowdown in its growth after a significant expansion between 1930 and 1932 (when it grew from six to twenty ‘services’). While syphilimetry centres had been established in nine colonies by 1932 – including Madagascar, Cochinchina, French Indies (Pondicherry), Senegal, Guyana, Martinique, Tahiti, Cameroon, and Algeria^102^ – sixteen had a syphilimetry laboratory by 1939. To those already mentioned were added Cambodia, Guadeloupe, Syria, Congo, Chad, New Caledonia, Reunion, and Ivory Coast. The institute reached its maximum colonial extension just before the Second World War. In the context of financial difficulty and decline, overseas development allowed it to maintain a part of its influence with the political authorities and the media.

One indication of the IP’s colonial turn was the changing involvement of its founder. Until the mid-1930s, Vernes had personally remained relatively distant from colonial issues. Although he promoted overseas activities from within France and participated in improving colonial equipment, he left the responsibility of handling colonial affairs to other, more competent personalities at the Institut. Thus, while Nguyen Van Tung and Trautmann were the representatives of the Institute’s establishment on the ground, Léger and Sorel were promoting the benefits of syphilimetry within their networks, especially the *Société de Pathologie Exotique.* However, at the end of 1938, Vernes was personally involved in an inspection mission in AOF [*Afrique-Occidentale Française* – French West Africa], entrusted by the Minister of Colonies. Accompanied by Trautmann, it was an extensive tour passing through Dakar, Bamako, Kindia, and Conakry between 2 December 1938 and 2 January 1939.^103^ The inspection mission did not only concern venereal diseases but all the endemic diseases affecting this region of the empire, such as trypanosomiasis, yellow fever, malaria, and leprosy. In concluding their report, Vernes and Trautmann inserted themselves into the doctrine that dominated French colonial health policy during the inter-war period: ‘The primordial obligation is to promote Black natality, and robust individuals, despite all the scourges that gather upon its head.’^104^ The authors add to their report a ‘Note on the reorganisation and development of syphilimetry in AOF (Vernes Method).’ The results in Western Africa were poor and disappointing for the IP. In 1939, only three Vernes machines were in operation in French West Africa: two in Dakar and one in Ouagadougou. Moreover, Vernes and Trautmann recognised that ‘the specialised personnel is lacking’. In the end, the authors again expressed pious wishes and announced that they were planning to install seven new devices at an undetermined future time in order to spread syphilimetry in the colonies of Ivory Coast, Guinea, Niger, Sudan, and Dahomey, where it was still completely absent. Vernes offered to send a trainer to Dakar for six months and suggested the imposition of a one-month training period in the IP laboratories for all pharmacists of the Colonial Troops destined for AOF.^105^ These modest proposals, at a time of crisis for the IP, reflected a feeling of resignation regarding the lack of progress since the first syphilimetry laboratory had been established in Dakar in 1928.

As a skilful communicator, Vernes took advantage of this mission to publicise its work, as he had been used to doing during his activities in France. According to him, the press had always been ‘faithful’ to the Institute.^106^ In the colonial press, the mission was reported, for example, by the newspaper *Paris-Congo* in the 11 December 1938 issue.^107^ In the French metropolitan press, *La Petite Gironde* reported on the mission in two articles, one before his departure and another upon his return.^108^ After returning to France, Vernes’ declarations to the press contrast with his observations in the field and demonstrate that he was engaged in a promotional effort: ‘We have travelled through Senegal, Sudan, and Guinea. In the centres where syphilimetric methods are applied, great progress has been made, thanks to the continued energy of a staff that provides the best example of self-sacrifice for the service of humanity.’ In the same statement, he added: ‘If we can say that we have seen unexpected results, we must recognise that much remains to be done.’^109^

The late 1930s was a pivotal period for the IP. While its expansion in the colonies partly masked its decline in France, its presence was uneven across the empire. Efforts to promote syphilimetry and the Vernes method in the poorly resourced colonies were not only strategic but also reflected the IP’s inability to establish itself as a genuine alternative in the global fight against syphilis.

## The failure of the expansion of the Vernes method in the colonies

Despite the introduction of syphilimetry in some parts of the empire, the colonial enterprise of the Institut Prophylactique failed on a global scale. On the one hand, the Vernes method hardly convinced most physicians working in the colonies or actively connected with tropical medicine. Some medical practitioners and health officers were even particularly hostile to implementing a treatment protocol considered to be out of touch with the realities of the field. On the other hand, if the colonial administration appreciated the material and human support provided by the IP concerning testing, the use of syphilimetry was largely diverted from its original purpose in the colonial context. For the French State and its colonial administration, if the help offered was welcome in the control of venereal diseases against a background of shortage and insufficient budgets, there was no effort to implement the Vernes method itself (i.e. the complete care of syphilitic patients and their follow-up by syphilimetry following the eight-month rule).

It was within a broader debate on the therapeutic strategy that should be adopted for the treatment of native syphilitics that the IP tried to convince colonial and tropical physicians.^110^ This discussion occurred in particular within the *Société de Pathologie Exotique*, of which Léger was a member. In 1931, he initiated a debate on the prevailing practices in the control of venereal diseases in the colonies, namely the massive use of arsenobenzenes,^111^ new arsenic-based drugs developed since the introduction of 606 by Paul Ehrlich.^112^ The Society of Exotic Pathology was mainly composed of practitioners practicing (or having practiced) in the colonies, who had to be convinced to adopt the Vernes method. If most of them recognised the merits of the method advocated by the IP, they were almost unanimous in judging its generalised application in the colonies as utopian. On the one hand, it would require an extremely high cost in human and financial resources,^113^ in total contrast with the systemic precariousness of the colonial health system. On the other hand, even if these resources were allocated, it would take years, if not decades, to make them accessible to all the populations of the empire. Moreover, those who supported the IP’s colonial project recognised the material impossibility of covering all the ultramarine territories in the short term, while being convinced that this was an achievable horizon. Nevertheless, while waiting for this situation to evolve, they considered that treatments unsupported by a serious serological follow-up should be abandoned, especially when practiced with arsenobenzenes, considering the iatrogenic effects induced by their use. According to them, the most reasonable solution would be either to use alternative, less iatrogenic (even if also less effective) drugs, such as mercury, or therapeutic abstinence (i.e. the total absence of treatment).^114^ This astonishing proposal was based on the idea – shared by a significant proportion of French proponents of syphilimetry – that syphilis had a relatively benign pathology among African and Asian populations. Thus, while promising the extinction of syphilis in the indefinite future, the doctors associated with the IP were unable to propose valid alternatives in the shorter term.

**Figure 4.**
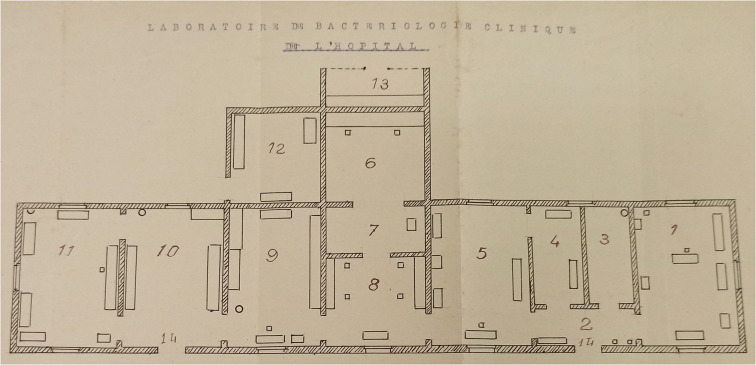
Clinical bacteriology laboratory at the Dakar Hôpital Principal (1928). The Vernes laboratory occupies rooms 4 and 5. ANS, 2G 28, no. 17. (photograph: the author.)

Implementing the Vernes method in the colonies was also a failure regarding its application in the field. The existence of a Vernes laboratory did not necessarily mean the establishment of long-term treatment and regular serological follow-up for each patient. Sometimes, a so-called Vernes laboratory only refers to the presence of a photometer in a colonial health facility. While a few colonial institutes applied the Vernes method, syphilimetry centres usually only contributed to the routine work of the laboratories hosting them. In the context of limited resources, this provided valuable material support. In Senegal, for instance, a photometer had been installed in a modest room in the bacteriology laboratory of Dakar’s *Hôpital Principal* in 1928 (see Figure 4), and the ‘Vernes reaction’ was the only one used during that year to detect syphilis (476 tests).^115^ The laboratory was directed by Dr. Golovine,^116^ who recognised – as did the majority of physicians – the value of syphilimetry as a screening test but was sceptical about the possibility of implementing the Vernes method itself in the colonies. In addition to material and economic arguments, he also based his opinion on racist considerations, considering that the level of ‘social education’ of the colonised populations would be insufficient for a method requiring long-term treatment. According to him, ‘For the syphilimetric method to be truly applicable to the colonies, it will first be necessary to raise the social level of the savage populations.’^117^ In Dakar, the Vernes reaction was used throughout the 1930s by the Hôpital Principal. However, this institution was soon supplanted in syphilis screening by the Pasteur Institute, which preferred to use other serological reactions. In 1942, while 590 Vernes tests were carried out in Dakar by the Hôpital Principal, the Institut Pasteur performed 6,153 tests using other reactions (e.g. Kahn and Hecht-Levaditi).^118^

The arrival of the IP on the colonial scene came at an opportune time, in terms of resource needs, for the colonial administration. The Agreement of Brussels, ‘respecting Facilities to be given to Merchant Seamen for the Treatment of Venereal Diseases’, signed on 1 December 1924, committed the French government to make screening and treatment facilities available to seafarers in the main trading ports of the colonial empire. In this context, the IP offered valuable material support, but its treatment methods were not seriously considered. In the port of Dakar, for instance, a dispensary had been set up in compliance with the 1924 Agreement,^119^ and the Vernes laboratory at the city’s Hôpital Principal was asked to participate in the testing effort after its creation. Frank Cazanove, head of the Health Service of the Dakar District and an enthusiastic supporter of syphilimetry, nevertheless recognised the impossibility of applying the Vernes method. However, he opposed the idea that the level of civilisation of the colonised population was insufficient to be treated following the Vernes method. In his view, ‘the working conditions of the workers at the Port of Dakar made it impossible for them to be treated in this way’.^120^ He also criticised his colleagues’ attitude towards syphilitic patients, identifying this as a cause of their non-adherence to treatment: ‘The native is not just a vein into which a needle is inserted. He has a name, which indicates a race, a country, customs, habits, and rites; talking to him knowingly, asking him for some details, is extremely appreciated by him.’^121^

Although syphilimetry was introduced in some of the colonies in the 1930s, the Vernes method – intended to achieve a complete cure – struggled to get off the ground. From the outset, the photometer was used in the colonies as a new testing tool to detect infected patients, but this had no real influence on the approach to treatment. Furthermore, the history of the IP in the French colonial empire was also closely linked to the conditions under which it could be implemented (or not) at the local scale. Under the impetus of Trautmann and Nguyen Van Tung, real syphilimetry programs were developed in Madagascar and Saigon. However, most doctors in the health services of the rest of the colonial empire seemed reluctant to see it as anything other than a new screening facility.

## Conclusion

The history of the Institut Prophylactique during the inter-war period is one of failure. At a time when the ‘venereal peril’ was presented as a health policy priority, Vernes’ project offered a testing device (the photometer), a ‘rational’ treatment method (the ‘Vernes method’), and a strategy designed to lead to the eradication (‘extinction’) of syphilis. Despite limited success, syphilimetry and the IP were pivotal in the French anti-venereal effort. Both in metropolitan France and in part of the colonies, syphilimetry significantly contributed to the mobilisation against syphilis in the 1920s–1930s. However, history has forgotten the IP, overshadowed by two more successful actors: the Institut Fournier and the Institut Pasteur. Its study offers a different view on this time, which is essential for understanding the complex history of the ‘fight’ against syphilis in the inter-war period. Furthermore, the history of Arthur Vernes’ Institute in France cannot be separated from its colonial context.

Examining the rise and decline of the IP during the inter-war years not only sheds light on the history of an alternative project but also illuminates the multiple genealogies of the idea of eradication. Although most often associated with the post-Second World War period – through campaigns orchestrated by the WHO or colonial administrations, and later with the eradication of smallpox^122^ – eradication is part of a longer history dating back to the early twentieth century. The imperial origins of the modern idea of eradication have been highlighted by Nancy Leys Stepan, who traces it back to American campaigns against yellow fever and the development of the Rockefeller Foundation.^123^ She identified several characteristics of a new model of public health for the control and elimination of infectious diseases, notably ‘its biomedical character’ (thanks to bacteriology, which allowed the identification of a tangible enemy) and ‘its single-disease focus’. Arthur Vernes’ project aligned with these two characteristics. However, Stepan also notes ‘the organisation of the control methods as a “campaign” set up independently of, or outside, existing public health services’.^124^ On this point, the IP differed. Unlike its opponents – who, without seeking eradication, advocated mass treatment campaigns on the model of the so-called ‘Jamot Mission’ against trypanosomiasis (sleeping sickness) in Cameroon from the 1920s onwards^125^ – its project did not involve such campaigns against syphilis. Vernes aimed to make his method the international standard for the diagnosis and treatment of syphilis, envisioning the eventual ‘extinction’ of the disease through sustained investment.

Until decolonisation, colonial health policies were dominated by mass medicine and strategies that focused on interrupting transmission chains rather than on curing individual patients. Such approaches were taken for trypanosomiasis, leprosy, yaws, and syphilis. But there were also alternative projects at the time. Vernes’ aim was to treat syphilitics from all over the empire in the same way as patients in France. Most of the IP’s members were opposed to the generalised view that colonised populations were insufficiently civilised to undergo complete treatment and that they were responsible for not being entirely cured – of syphilis, as for other pathologies. Of course, it is absolutely not to suggest that they were exemplars of what might have constituted a ‘good’ colonial medicine. The members of the IP were themselves deeply imbued with the racial prejudices of their time. However, they articulated a distinctly different approach – less coercive than the prevailing model of mass medicine – which they conceived as a possible path towards more effectively improving the health of the empire’s populations. In other words, a different approach intended to support the success of the colonial project – as well as the success of the IP.

Although the IP failed in its plans to eradicate syphilis, its ideas spread and were discussed or implemented locally, as in Madagascar and Indochina. Arthur Vernes, his colleagues, and syphilimetry contributed to reshaping the control of infectious diseases during the inter-war years. It is a striking example of why failures, alternative, and unsuccessful projects are central to the history of health and its narratives. Choices that came to dominate colonial healthcare practices were shaped by alternative proposals, whether alongside or in opposition to them.

## Footnotes

1 This was the idea that syphilis could be inherited from a syphilitic parent, even beyond the first generation. Alain Corbin , ‘L’hérédosyphilis ou l’impossible rédemption. Contribution à l’histoire de l’hérédité morbide’, Romantisme, 31 (1981), 131–5011633079 .

2

Virginie De Luca Barrusse , ‘Natalisme et hygiénisme en France de 1900 à 1940. L’exemple de la lutte antivénérienne’, Population, 64, 3 (2009), 531–60; Virginie De Luca Barrusse , Population en danger! La lutte contre les fléaux sociaux sous la Troisième République (Bern: Peter Lang, 2013); Jean-Yves Le Naour , ‘Sur le front intérieur du péril vénérien (1914–1918)’, Annales de démographie historique, 103, 1 (2002), 107–2017323536 ; Claude Quétel , Le mal de Naples (Paris: Seghers, 1986); Yann Ardagna and Benoît Pouget (eds), La syphilis Itinéraires croisés en Méditerranée et au-delà XVI^e^-XXI^e^ siècles (Aix-en-Provence: PUP, 2021); Christian Bonah , Guillaume Linte and Alexandre Wenger , Maladies infectieuses sans fin. Le cas de la syphilis pour penser la mobilisation-démobilisation prophylactique (XX^e^-XXI^e^ siècle) (Geneva: Georg, 2022). See also the special issue of the *Annales de Démographie Historique*, ‘Syphilis and STIs. Epidemiology and the Use of Figures: From the “Venereal Peril” to HIV’, 148, 2 (2024).

3

Myron Echenberg , ‘“Faire du nègre”. Military aspects of population planning in French West Africa, 1920–1940’, in D.D. Cordell and J.W. Gregory (eds), African Population and Capitalism: Historical Perspectives (Boulder: Westview Press, 1987). The offensive French term ‘nègre’ is kept uncensored here to reflect – and not downplay – the racist character of colonial medicine.

4 Although the IP is sometimes mentioned in historical works, its action in itself is not discussed. Only one article has been devoted to the history of the IP in recent years: Gérard Tilles , ‘En finir avec la syphilis. L’ambition inaboutie d’Arthur Vernes’, Annales de Dermatologie et de Vénéréologie, 147, 1 (2020), 57–73 31864763 10.1016/j.annder.2019.11.003.

5

Aro Velmet , Pasteur’s Empire: Bacteriology and Politics in France, its Colonies, and the World (New York: Oxford University Press, 2020).

6

Megan Vaughan , Curing Their Ills: Colonial Power and African Illness (Stanford: Stanford University Press, 1992), 151 .

7

Megan Vaughan , ‘Syphilis in Colonial East and Central Africa, the social construction of an epidemic’, in T. Ranger and P. Slack (eds), Epidemics and Ideas: Essays on the Historical Perception of Pestilence (Cambridge: Cambridge University Press, 1992), 269–302 .

8

Philippa Levine , Prostitution, Race & Politics. Policing Venereal Disease in the British Empire (New York: Routledge, 2003), 1–2 .

9

Katherine Paugh , ‘Yaws, Syphilis, Sexuality, and the Circulation of Medical Knowledge in the British Caribbean and the Atlantic World’, Bulletin of the History of Medicine, 88, 2 (2014), 225–5224976161 10.1353/bhm.2014.0029.

10

Samuël Coghe , Population Politics in the Tropics: Demography, Health and Transimperialism in Colonial Angola (Cambridge: Cambridge University Press, 2022), 178–205 .

11

Philip J. Havik , ‘Public Health, Social Medicine and Disease Control: Medical Services, Maternal Care and Sexually Transmitted Diseases in Former Portuguese West Africa (1920–63)’, Medical History, 62, 4 (2018), 504 10.1017/mdh.2018.44PMC615864830191784.

12

Anne Cornet , Politiques de santé et contrôle social au Rwanda: 1920–1940 (Paris: Karthala, 2011).

13

João Dinis Sousa , Philip J. Havik , and Anne-Mieke Vandamme , ‘Sexually Transmitted Infections, Their Treatment and Urban Change in Colonial Leopoldville, 1910–1960’, Medical History, 65, 2 (2021), 178–96.

14

Philip W. Setel , Milton Lewis and Maryinez Lyons (eds), Histories of Sexually Transmitted Diseases and HIV/AIDS in Sub-Saharan Africa (Westport, CT: Greenwood Press, 1999).

15

Mor Ndao , ‘Les prises en charge des pathologies vénériennes par le pouvoir colonial au Sénégal, 1850–1960. Éclairages et enseignements pour le sida’, Annales de la Faculté des Lettres et Science Humaines, 39, B (2009), 40–68 ; Charles Becker and René Collignon, ‘A history of sexually transmitted diseases and AIDS in Sénégal: difficulties in accounting for social logics in health policy’, in P.W. Setel, M. Lewis and M. Lyons (eds), *op. cit.* (note 14), 65–96.

16

Christelle Taraud , La prostitution coloniale. Algérie, Tunisie, Maroc (1830–1962) (Paris: Payot, 2003); Isabelle Tracol-Huynh , ‘Between Stigmatisation and Regulation: Prostitution in Colonial Northern Vietnam, Culture, Health & Sexuality’, Culture, Health & Sexuality, 12, supp. 1 (2010), S73–S87 10.1080/1369105100370656120364443; Isabelle Tracol-Huynh , ‘Prostitution in Colonial Hanoi (1885–1954)’, in M.R. García , L. Heerma van Voss and E. van Nederveen Meerkerk (eds), Selling Sex in the City: A Global History of Prostitution, 1600s–2000s (Leiden: Brill, 2017) .

17

Kalala Ngalamulume , ‘Le péril vénérien. L’État colonial français et la sexualité à Saint-Louis du Sénégal, 1850–1920’, in J.-P. Bado (ed.), Les conquêtes de la médecine moderne en Afrique (Paris: Karthala, 2006); Ndao, *op. cit.* (note 15).

18

Idrissou Alioum , ‘Prostitution et lutte contre les maladies vénériennes au Cameroun, 1923–1964: discours et pratiques’, Kaliao, 2, 4 (2010), 123–41.

19

Yacouba Banhoro , ‘Essai historique sur la prostitution et la prophylaxie antivénérienne dans la Haute-Volta coloniale’, Annales de l’Université de Ouagadougou, 20, A (2015), 179–212 .

20

Hannah-Louise Clark , ‘Civilization and Syphilization: A Doctor and His Disease in Colonial Morocco’, Bulletin of the History of Medicine, 87, 1 (2013), 86–114 23603530 10.1353/bhm.2013.0003.

21 Marie Brualla Challet, ‘Existe-t-il une “syphilis tahitienne” ? Études historiques et épidémiologiques sur les spécificités de la syphilis à Tahiti (1860–1960)’, in Ardagna and Pouget, *op. cit.* (note 2), 393–406.

22

Guillaume Linte , ‘
*Syphilis exotique.* Comment la médecine a fait de la syphilis une maladie dermatologique “bénigne” durant la colonisation (France, 1900–1940)’, Revue Suisse d’Histoire, 74, 3 (2024), 317–33.

23 Although this term may seem to have a particular link with the colonial context, this is not the case. *Blanchiment* was used to express the disappearance of syphilis symptoms in France in the nineteenth and twentieth centuries, regardless of the patient’s origin. However, although this expression is not associated with explicit racial considerations in the sources I have studied so far, it may have been different in other periods, regions, or contexts not covered by my study.

24

Guillaume Linte , ‘Syphilis, *blanchiment* and French Colonial Medicine in Sub-Saharan Africa During the Interwar Period’, Medical History, 67, 4 (2023), 313–510.1017/mdh.2023.29PMC1061669437828844.

25

Helen Tilley , Africa as a Living Laboratory: Empire, Development, and the Problem of Scientific Knowledge, 1870–1950 (Chicago and London: University of Chicago Press, 2011).

26 An approach, for example, that was developed by Antonio García Belmar and José Ramón Bertomeu-Sánchez , ‘L’Espagne fumigée. Consensus et silences autour des fumigations d’acides minéraux en Espagne, 1770–1804’, Annales Historiques de la Révolution Française, 383, 1 (2016), 177–202 .

27 Linte, *op. cit.* (note 24), 313–8.

28 On this issue, see, for instance: Esoavelomandroso Faranirina ‘Résistance à la médecine en situation coloniale: la peste à Madagascar’, Annales. Économies, Sociétés, Civilisations, 36, 2 (1981), 168–9011631509; Kalle Kananoja , Healing Knowledge in Atlantic Africa: Medical Encounters, 1500–1850 (Cambridge: Cambridge University Press, 2021); Laurence Monnais , The Colonial Life of Pharmaceuticals: Medicines and Modernity in Vietnam (Cambridge: Cambridge University Press, 2019), 233–53.

29

Rita Headrick , Colonialism, Health and Illness in French Equatorial Africa, 1885–1935 (Atlanta: African Studies Association Press, 1994); Coghe, *op. cit.* (note 10); Guillaume Lachenal , Le médicament qui devait sauver l’Afrique. Un scandale pharmaceutique aux colonies (Paris: La Découverte, 2014); Guillaume Lachenal , ‘A Genealogy of Treatment as Prevention (TasP): Prevention, Therapy, and the Tensions of Public Health in African History’, in T. Giles-Vernick and James L.A. Webb Jr. (eds), Global Health in Africa: Historical Perspectives on Disease Control (Athens: Ohio University Press, 2013), 70–91 .

30

Arthur Vernes , Atlas de syphilimétrie. Les conditions expérimentales de l’extinction de la syphilis (Paris: Pradier, 1920).

31 Tilles, *op. cit.* (note 4), 57.

32

Arthur Vernes , Les signes humoraux de la syphilis (Paris: J.-B. Baillière et fils, 1913).

33 On this topic, see: Ilana Löwy , ‘Les “Faits scientifiques” et leur public: l’histoire de la détection de la syphilis’, Revue de synthèse, 116, 1 (1995), 27–54 11624865 10.1007/BF03181266; Ilana Löwy , ‘“A River That Is Cutting Its Own Bed”: The Serology of Syphilis Between Laboratory, Society and the Law’, Studies in History and Philosophy of Biological and Biomedical Sciences, 35, 3 (2004), 509–24.

34 Arthur Vernes published several articles on Wassermann reaction, for instance with the influential dermato-venereologist Edouard Jeranselme: ‘De la reaction Wassermann appliquée au traitement de la syphilis’, *Paris-Médical*, 5 (11 March 1912), 329–36.

35 The tests were conducted monthly.

36

Arthur Vernes , ‘La lutte contre la syphilis. Vue d’ensemble’, Archives de l’Institut Prophylactique, I, 1 (1929), 3 .

37
*Ibid.*, 20.

38 Vernes, *op. cit.* (note 30); Arthur Vernes, *Travaux et Publications de l’Institut Prophylactique*, Fascicule 1[–5] (Paris: Maloine, 1922[–1926]).

39

Arthur Vernes , ‘Au seuil de notre deuxième année de publication’, Archives de l’Institut Prophylactique, II, 1 (1930), 6.

40 For more details on the support Vernes received at the founding of the Institut Prophylactique, see Tilles, *op. cit.* (note 4), 63–4.

41 Alexandre Wenger, ‘Littérature et syphilis. Le roman de prévention antivénérien (1890–1910)’, *Arts et Savoirs* [online], 18 (2022); Alexandre Wenger, ‘Cas médical et prévention antivénérienne au tournant du XX^e^ siècle: Les Avariés d’Eugène Brieux’, *Fabula, Les colloques, Littérature. et écritures du cas*, online: http://www.fabula.org/colloques/document7041.php.

42 2,000,000 francs from the State and 900,000 francs from French *départements* and municipalities. Arthur Vernes, ‘Rapport à l’Assemblée Générale de l’Institut Prophylactique (21 décembre 1932)’, *Archives de l’Institut Prophylactique*, V, 1 (1933), 11–19.

43 Jacques Dumont, ‘Prise en charge des corps. Profil du médecin colonial à partir de quelques itinéraires (Antilles, fin XIX^e^ siècle-années 1960)’, *Outre-Mers*, 398–399 (2018), 68–70.

44

Marcel Léger , ‘La syphilis dans les Colonies françaises. Les avantages de la méthode syphilimétrique Vernes dans la lutte contre l’endémie’, Revue de médecine et d’hygiène tropicale, 17 (1925), 116–31.

45

Nguyen Van Tung , ‘Fonctionnement de l’Institut Prophylactique de Saïgon en 1926, 1927, 1928’, Archives de l’Institut Prophylactique, II, 1 (1930), 15–22 ; René Trautmann , ‘Notes sur la syphilis à Madagascar’, Archives de l’Institut Prophylactique, I, 4 (1929), 303–31.

46 ‘Services de l’Institut Prophylactique’, *Archives de l’Institut Prophylactique*, II, 3 (1930), 356.

47

René Trautmann , ‘Aperçu de la lutte entreprise contre la syphilis à Madagascar’, Archives de l’Institut Prophylactique, III, 4 (1931), 519–20.

48

Madagascar, Cochinchina , Pondicherry, Senegal , Guyana, Martinique , Tahiti, Cameroon , Guadeloupe, Syria . ‘Service de l’Institut Prophylactique’, Archives de l’Institut Prophylactique, VII, 1 (1935), 9–10 .

49 ‘Rapport à l’Assemblée Générale de l’Institut Prophylactique (20 décembre 1929)’, *Archives de l’Institut Prophylactique*, II, 1 (1930), 7.

50

Arthur Vernes , ‘Des moyens à employer pour lutter contre la syphilis aux colonies’ [Extract from a speech given to the Académie des Sciences Coloniales at a session held at the Institut Prophylactique on 7 February 1930] Archives de l’Institut Prophylactique, II, 3 (1930), 293 .

51
*Ibid.*, 296.

52
*Ibid.*, 297.

53

Marcel Léger , ‘La lutte contre la syphilis chez les originaires des colonies’ [Report presented at the 11 July 1930 meeting of the Consultative Commission for the Prophylaxis of Venereal Diseases in the Colonies], Archives de l’Institut Prophylactique, II, 3 (1930), 299 .

54
*Ibid.*, 299–314.

55
*Ibid.*, 299–300.

56
*Ibid.*, 301.

57 ‘Rapport à l’Assemblée Générale de l’Institut Prophylactique (17 décembre 1931)’, *Archives de l’Institut Prophylactique*, IV, 1 (1932), 19.

58 Pierre Even and Marcel Leger, ‘La Guadeloupe et son redressement sanitaire (rapport de mission)’, *Archives de l’Institut Prophylactique*, IV, 2 (1932), 131–76.

59 Alfred Chaumel (real.), *Symphonie Malgache* (Synchro-Ciné: 1934).

60 Vernes, *op. cit.* (note 50), 294.

61 Marcel Léger, ‘La vie de l’Institut Prophylactique en 1930’, *Archives de l’Institut Prophylactique*, III, 3 (1931), 360.

62 Their nationalities are specified: ‘France: 200; other European countries: 39; South America: 19; Central America, Antilles: 6; North America: 3; Asia Minor and Persia: 7; Egypt: 2; China: 2’. *Ibid.*

63
*Ibid.*, 361–2.

64

Arthur Vernes , Robert Bricq , and René Trautmann , ‘Adaptation du matériel de syphilimétrie à la pratique coloniale’, Archives de l’Institut Prophylactique, II, 1 (1930), 69 .

65

Valérie Vignaux , ‘Les animateurs français et le Pathé-Baby ou des usages privés des images cinématographiques dans la France de l’entre-deux-guerres’, 1895. Mille huit cent quatre-vingt-quinze, 59 (2009), 83 .

66 Léger, *op. cit.* (note 53), 303.

67

Marcel Léger , ‘La vie de l’Institut Prophylactique en 1931’, Archives de l’Institut Prophylactique, IV, 3 (1932), 312 .

68 Tung, *op. cit.* (note 45), 15.

69 General Government of Indochina, General Inspection of Health and Medical Services. ‘Note on social hygiene in Indochina’, 1926, 4. French National Overseas Archives [hereafter: ANOM], AGEFOM 984.

70 In 1927, by its director Nguyen Van Tung and two auxiliary doctors, including Nguyên van Sang. *L’écho Annamite*, 12 January 1927.

71 The Hanoi Medical School opened its doors in 1902 and, as Laurence Monnais has demonstrated, was central to the process of professionalisation of the native physician in Vietnam during the first half of the twentieth century: Laurence Monnais-Rousselot, ‘Paradoxes d’une médicalisation coloniale. La professionnalisation du “médecin indochinois” au XX^e^ siècle’, *Actes de la recherche en sciences sociales*, 143, 3 (2002), 36–43. See also: Laurence Monnais-Rousselot , Médecine et colonisation. L’aventure indochinoise, 1860–1939 (Paris: CNRS Éditions, 1999).

72 His dissertation was published in 1924: Nguyen Van Tung , Le pian dans l’Indochine française (Paris: Éditions de la vie universitaire, 1924).

73 ‘Discours de M. Le Fol, Gouverneur intérimaire, à la session ordinaire de 1926 du Conseil Colonial de Cochinchine’, *L’Écho Annamite*, 12 August 1926.

74 Léger, *op. cit.* (note 61), 349–51.

75 Letter from the Inspector General of Hygiene and Public Health to the Governor General of Indochina [on the fight against venereal diseases], 11 October 1933, 3. ANOM, GGI CM 1599.

76 Decree of 2 December 1933, concerning the establishment in Indochina of a Commission for the fight against venereal diseases. ANOM, RST NF 3856.

77 Commission for the prophylaxis of venereal diseases: Minutes of the 1st meeting (8 January 1934). ANOM, RST NF 1856.

78 Decree of 2 December 1933, *op. cit.* (note 76). ANOM, RST NF 3856.

79 Letter no. 2285-s/s dated 16 March 1932. Quoted in: Commission for the prophylaxis of venereal diseases: Minutes of the 1st session (8 January 1934), 3. ANOM, RST NF 3856.

80
*Ibid.*

81 Commission for the prophylaxis of venereal diseases: minutes of the 2nd meeting (2 May 1934), 12. ANOM, RST NF 3856.

82
*Ibid.*, 27.

83 ‘Summary of the current situation in the fight against venereal diseases’, by De Raymond. 10 August 1934. ANOM, RST NF 3856.

84
*Ibid.*

85 Commission for the prophylaxis of venereal diseases: minutes of the 3rd meeting (27 September 1934), 4. ANOM, RST NF 3856.

86
*Ibid.*, 5.

87 Letter from Marius Moutet (Minister for the Colonies) to the Governor General of Indochina, 12 December 1936. ANOM, RST NF 3856.

88 Letter from Dr. P. Hermant, Inspector General of Hygiene and Public Health, to the Governor General of Indochina, 20 February 1937. ANOM, RST NF 3856.

89
*Ibid.*

90 According to the census taken on 1 July 1936. Annam had 5,656,000 inhabitants, Cambodia 3,046,000, and Laos 1,012,000. Henri Bunle , ‘Chronique de démographie’, Journal de la société statistique de Paris, 78 (1937), 361 .

91 All data are taken from: ANOM, RST NF 3856, Letter from Dr. P. Hermant, *op. cit* (note 88).

92 ‘Enseignement par le film’, *L’Écho Annamite*, 3–4 April 1929, 2.

93 Nguyen van Tung, ‘Le péril vénérien’, *L’Echo Annamite*, 8–9 October 1941, 4.

94 ‘Note from the Board of Directors of the Institut Prophylactique for the fight against syphilis. To the attention of those who hold the destiny of the Fatherland in their hands’. French National Academy of Medicine [hereafter: ANM], AMP Carrel 14-2, no. 3, 3. The document is signed by seven members of the Board of Directors: Alexandre Millerand, A. Lacroix, Gabriel Bertrand, A. Gosset, M. Triffeneau, Jacques Trefouel, and Arthur Vernes – who is almost certainly the author of the document.

95 Letter of Arthur Vernes to 24 October 1941, addressed to Henri Gangardel. ANM, AMP Carrel 14-2, no. 5, 3.

96 350,559 consultations in 1933; 332,043 in 1934; 310,199 in 1935; 280,448 in 1936; 269,999 in 1937; 252,229 in 1938; and 224,347 in 1939. ANM, AMP Carrel 14-2, no. 5, 3.

97 Löwy, *op. cit.* (note 33, 1995); Pauline M.H. Mazumdar (2003), ‘“In the Silence of the Laboratory”: The League of Nations Standardizes Syphilis Tests’, Social History of Medicine, 16, 3 (2003), 437–59.

98 At the maternity hospital of Lausanne, for example. Archives Cantonales Vaudoises (Lausanne), Obstétrique, K VIIIe 227–276. See also Mathilde Le Vu , Mario Cortina-Borja , Jonathan C.K. Wells , ‘Evaluating the Concept of Brain Sparing in a High Income Setting, Using Historical Records of Maternal Influenza or Syphilis Infection’, American Journal of Human Biology, 36, 11 (2024), e24143 39180148 10.1002/ajhb.24143.

99

Édouard Jeanselme , Traité de la Syphilis. I. Histoire de la syphilis (Paris: G. Doin, 1931), 398–9.

100 ANM, AMP Carrel 14-2, no. 5, 4.

101
*Ibid.*, 9.

102 Here I’m including Algeria in the colonial domain, even though it was officially a territory divided into French *départements* and counted with the metropolitan centres by the IP.

103 ‘The mission, after crossing Guinea and stopping for a few days in Conakry, embarked for France on 2nd January on the Paquebot Foucauld’. Extract from an anonymous note from 1939: ‘Guinée. The work of the Vernes-Trautmann mission’. ANOM, AGEFOM 384. Arthur Vernes and René Trautmann , ‘Extrait d’un rapport à M. le Ministre des colonies sur une mission en Afrique Occidentale Françaises’, Archives de l’Institut Prophylactique, XI, 1 (1939), 39–44 .

104
*Ibid.*, 42.

105
*Ibid.*, 43–4.

106 ANM, AMP Carrel, 14-2, no. 5. Letter of Vernes, *op. cit.* (note 95), 8.

107 Jean Mani, ‘La Science Française au service de l’Afrique. Ce que vont faire en A.O.F. les docteurs Vernes et Trautmann’, *Paris-Congo*, 11 December 1938. Found in ANOM, AGEFOM 384.

108 ‘Chargés de mission en Afrique le docteur Vernes et le docteur Trautmann se sont embarqués, samedi, à bord du Foucauld’, *La Petite Gironde*, 4 December 1938; ‘Chargés de mission médicale en Afrique par le ministère des colonies, les docteurs Vernes et Trautmann sont rentrés en France à bord du Foucauld’, *La Petite Gironde*, 13 January 1939.

109
*Ibid.*, 13 January 1939.

110 Linte, *op. cit.* (note 24), 315–22.

111

Marcel Léger , ‘Les arsénobenzènes aux Colonies: leur emploi raisonné et dûment contrôlé’, Bulletins de la Société de pathologie exotique et de ses filiales de l’Ouest africain et de Madagascar, 24 (1931), 394–406 .

112

Deborah Neill , ‘Paul Ehrlich’s Colonial Connections: Scientific Networks and Sleeping Sickness Drug Therapy Research, 1900–1914’, Social History of Medicine, 22, 1 (2009), 70 .

113

S. Golovine , ‘Observation sur le traitement de la syphilis colonies’, Bulletins de la Société de pathologie exotique et de ses filiales de l’Ouest africain et de Madagascar, 24 (1931), 641–3.

114

Marcel Léger , ‘Considérations sur le traitement de la syphilis chez les Indigènes de nos colonies’, Bulletins de la Société de pathologie exotique et de ses filiales de l’Ouest africain et de Madagascar, 24 (1931), 438 . See also: François Sorel , ‘La syphilis chez les indigènes en Afrique Occidentale’, Archives de l’Institut Prophylactique, V, 1 (1933), 23–4.

115 Annual report of the Health Service 1928. Dakar district and dependencies. Senegal National Archives [hereafter ANS], 2G 28, no. 17, 18.

116
*Ibid.*, 14–15.

117 Golovine, *op. cit.* (note 113), 641.

118 Médecin-Général Ricou, ‘Venereal diseases in French West Africa’ [report]. ANS, 2G 42, no. 7, 18 [1943]. It’s worth noting that the Institut Pasteur systematically performed both reactions for each serum, i.e. 6,153 were carried out with the Kahn reaction and the same number with the Hecht-Levaditi reaction.

119 Ndao, *op. cit.* (note 15), 10–11.

120

Frank Cazanove , ‘Les enseignements de deux années de lutte anti-syphilitique au port de Dakar’, Archives de l’Institut Prophylactique, V, 1 (1933), 33–4.

121
*Ibid.*, 40.

122

Randall M. Packard , A History of Global Health. Interventions into the Lives of Other Peoples (Baltimore: Johns Hopkins University Press), 133–79; Lachenal, *op. cit.* (note 29, 2014), 57–100.

123

Nancy Leys Stepan , Eradication. Ridding the World of Diseases Forever? (London: Reaktion Books Ltd, 2011), 35–103 .

124
*Ibid.*, 57–62.

125 A position defended by Eugène Jamot himself. Linte, *op. cit.* (note 24), 315–8. On the ‘Jamot Mission’ and the control of trypanosomiasis, see: Jean-Pierre Dozon , ‘Quand les Pastoriens traquaient la maladie du sommeil’, Sciences Sociales et Santé, III, 3–4 (1985), 27–56 11635938; Jean-Paul Bado , Eugène Jamot, 1879–1937: Le médecin de la maladie du sommeil ou trypanosomiase (Paris: Karthala, 2011); Guillaume Lachenal , ‘Médecine, comparaisons et échanges inter-impériaux dans le mandat camerounais: une histoire croisée franco-allemande de la mission Jamot’, Canadian Bulletin of Medical History, 30, 2 (2013), 23–45 28155399 10.3138/cbmh.30.2.23.

